# Characterizing the Relapse Potential in Different Luminal Subtypes of Breast Cancers with Functional Proteomics

**DOI:** 10.3390/ijms21176077

**Published:** 2020-08-24

**Authors:** Tung-Yi Lin, Pei-Wen Wang, Chun-Hsun Huang, Pei-Ming Yang, Tai-Long Pan

**Affiliations:** 1 Department of Traditional Chinese Medicine, Chang Gung Memorial Hospital at Keelung, Keelung 20401, Taiwan; tungyi30@cgmh.org.tw; 2 Department of Medical Research, China Medical University Hospital, China Medical University, Taichung 40447, Taiwan; pwwang5105@hotmail.com; 3 Department of Cosmetic Science, Research Center for Food and Cosmetic Safety, and Research Center for Chinese Herbal Medicine, College of Human Ecology, Chang Gung University of Science and Technology, Taoyuan 33303, Taiwan; chuang@gw.cgust.edu.tw; 4 TMU Research Center of Cancer Translational Medicine, Taipei 11042, Taiwan; yangpm@tmu.edu.tw; 5 Graduate Institute of Cancer Biology and Drug Discovery, College of Medical Science and Technology, Taipei Medical University, Taipei 11042, Taiwan; 6 School of Traditional Chinese Medicine, Chang Gung University, 259 Wen-Hwa 1st Road, Taoyuan 33302, Taiwan; 7 Liver Research Center, Chang Gung Memorial Hospital, Taoyuan 33375, Taiwan

**Keywords:** breast cancer, luminal B subtype, estrogen receptor, human epidermal growth factor receptor 2, proteomics, network analysis

## Abstract

Poor prognosis due to the high relapse and metastasis rates of breast cancer has been particularly linked to the luminal B subtype. The current study utilized MCF-7 and ZR-75-1 to investigate various luminal subtypes of breast cancers that have discrepant expressions in the estrogen receptor (ER) and human epidermal growth factor receptor 2 (HER2). Understanding of the differential protein profiles and the associated pathways could help alleviate the malignance and promote the long-term survival rate of breast cancer patients. Functional proteome tools were applied to comprehensively delineate the global protein alterations that reflect the varieties of biological features between the two subtypes. In this study, a total of 11 proteins with significant and meaningful changes were identified. These protein targets including PRX2, CK19, nucleophosmin and cathepsin D were mostly involved in cell differentiation or proliferation. Particularly, cathepsin D was highly expressed in the luminal B subtype. Moreover, the level of cathepsin-D was also upregulated in the clinical metastatic tissues. Accordingly, the RNA interference-mediated silencing of cathepsin D stimulated ER expression but suppressed the level of HER2. The knockdown of cathepsin D enhanced the level of ZO-1 and a remarkable decrease in N-cadherin was also detected. Again, the matrix metalloproteinases (MMP) activity was impaired under the cathepsin D abolishment. Collectively, this study represented a modality to explore novel relationships in a proteome complex and highlighted the functional roles of cathepsin D in treatment options for different subtypes of breast cancer.

## 1. Introduction

Breast cancer is the most common malignancy among women worldwide and the leading cause of female cancer-caused death. It is also a heterogeneous disease with large variations in its prognosis, indicating its apparent genetic and histopathological disparity. The classification of breast cancer is according to histological type, tumor grade, lymph node infiltration and the appearance of predictive markers, such as ER and HER2 [1,2,3,4]. Therefore, different subtypes of breast cancer have been used as a parameter to survey clinical outcomes [5,6,7]. Generally, Asian patients are younger and present lower frequencies of the luminal A and basal subtypes but higher frequencies of the luminal B and HER2-enriched subtypes, as compared to Western patients [8]. However, subjects diagnosed with the luminal B and HER2-enriched subtypes have a much lower survival rate and higher probability of relapse than those with the luminal A subtype. Furthermore, patients with luminal A subtype tumors have a long-term risk of distant metastatic disease, which is attenuated by tamoxifen administration, whereas patients with luminal B subtype tumors have an early risk of distant metastatic disease, and tamoxifen benefits reduce over time [9]. To investigate the molecular and proteomic profiling of breast cancer subtypes with different prognoses and treatment responses, we utilized the MCF-7 and ZR-75-1 cell lines to represent the luminal A and luminal B subtypes, respectively. Systemically revealing the differential molecular characteristics between these two subtypes of breast cancers could provide a novel insight into the prevention of breast cancer recurrence.

In spite of advances in breast cancer diagnosis and treatment, many treatments still fail due to disease progression, recurrence and an eventually reduced overall survival rate [10]. Particular cells possess higher tolerability to various therapies, and these cells yield the bulk of the tumor after reduction of the cell populations sensitive to the first-line treatment, thus leading to disease relapse. The relative resistance of breast cancer cells to anti-cancer agents may also result from a more efficient mechanism involved in the decrease of apoptosis and cell differentiation states (namely, EMT), as compared to other mammary cell types [11,12]. Given the significance of EMT in tumor initiation and metastasis, effective therapeutic strategies and novel drugs targeting EMT in breast cancer have become critical for improving the survival rate and quality of life for those already diagnosed with cancer relapse.

Numerous genomic and transcriptomic studies have been conducted to explore the molecular characteristics associated with the recurrence of breast cancer, yet it is difficult to elucidate correlations, as the transcriptome cannot perfectly insinuate those of the proteome [13,14,15]. In addition, a large amount of proteins and molecules are changed both in quantity and quality during the relapse of breast cancer, which reflects pathological abnormalities and disease progression [16]. Thereby, functional proteome methods combined with bioinformatics for data mining offer a feasible tool for high-throughput screening and differentially identifying protein targets that are connected to the pathogenesis and the pinpointing signaling pathways resulting in the deterioration of breast cancer. In the current study, we applied MetaCore^TM^ pathway software to extensively analyze the cellular interplay and associated molecular mechanisms behind the differences in protein levels.

Despite previous documents mentioning the possible pathology and mechanisms related to the relapse of breast cancer, no effort has been made to distinguish the discrepancy in global protein expression and the biological effects between the luminal A and luminal B subtypes. Our findings could bridge the current gap in therapeutic intervention and management strategies for breast cancer in a setting of ethnic diversity.

## 2. Results

### 2.1. Comparison of the Different Breast Cancer Cell Models Reflecting Breast Cancer Heterogeneity

**Figure 1:**
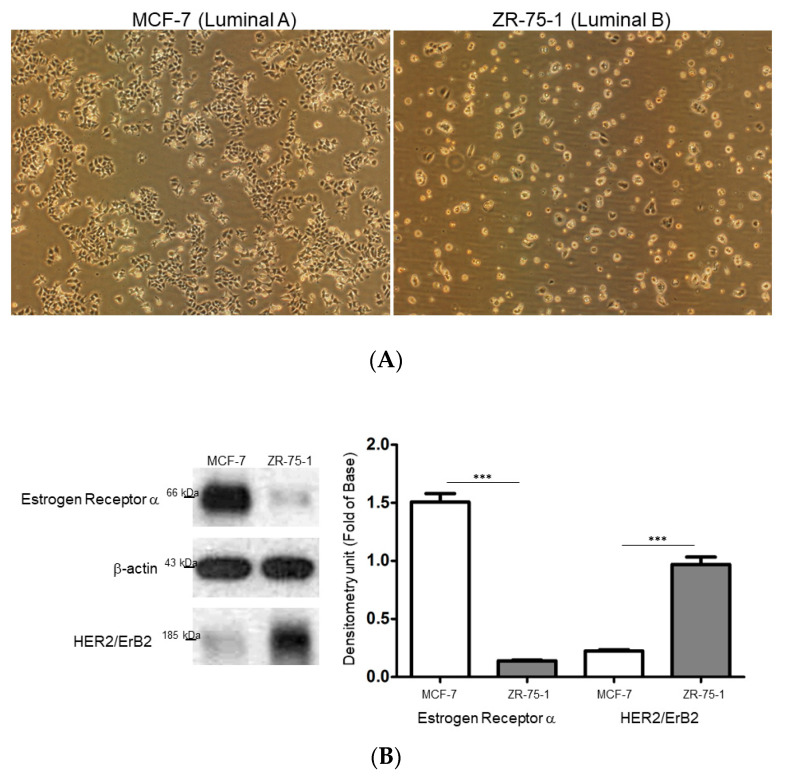
(**A**) Morphological comparison between MCF-7 and ZR-75-1 cells. Original magnification: 100×. (**B**) Evaluation of the levels of ER and HER2 in MCF-7 and ZR-75-1 cells. The quantified results were demonstrated by the bar chart and data were the mean ± SD of three independent experiments. β-actin was utilized as a loading control and (*** *p* < 0.001).

Since breast cancers consist of different tumor subtypes with various clinical characteristics, the ER-positive/HER2 negative, MCF-7 and ER-negative/HER2 positive ZR-75-1 breast cancer cell lines were applied to respectively represent the luminal A and luminal B subtypes in the current study. Based on morphological observations made under a microscope, the MCF-7 cells appeared to be more proliferative than the ZR-75-1 cells and showed growth in clusters with cell-to-cell connections similar to epithelial cells. In contrast, the ZR-75-1 cells had a rounder appearance, similar to cells with highly invasive potential (Figure 1A). At the same time, we evaluated the levels of ER and HER2 in the MCF-7 and ZR-75-1 cells using Western blot analysis. As expected, the MCF-7 cells showed a remarkable expression of ER and a quite low signal of HER2, whereas the ZR-75-1 cells had a high level of HER2 and a small amount of ER (Figure 1B). These differences in morphology and molecular features could explain the disparity in clinical results for various subtypes of breast cancer. 

### 2.2. Exploring Target Proteins with Functional Proteome Tools

**Figure 2:**
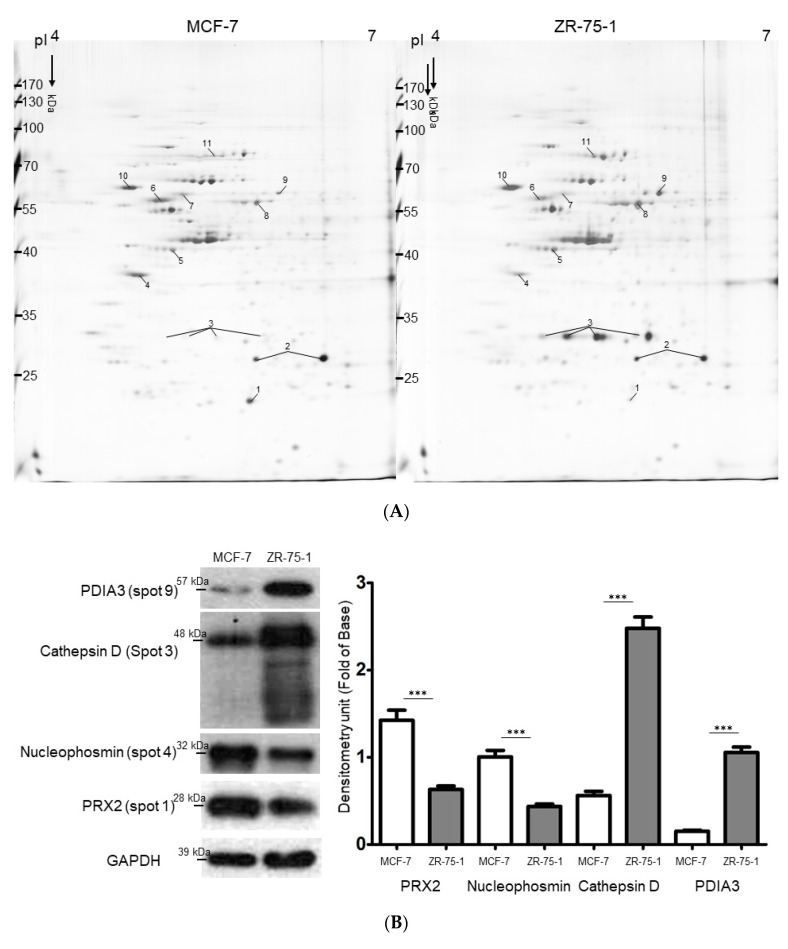

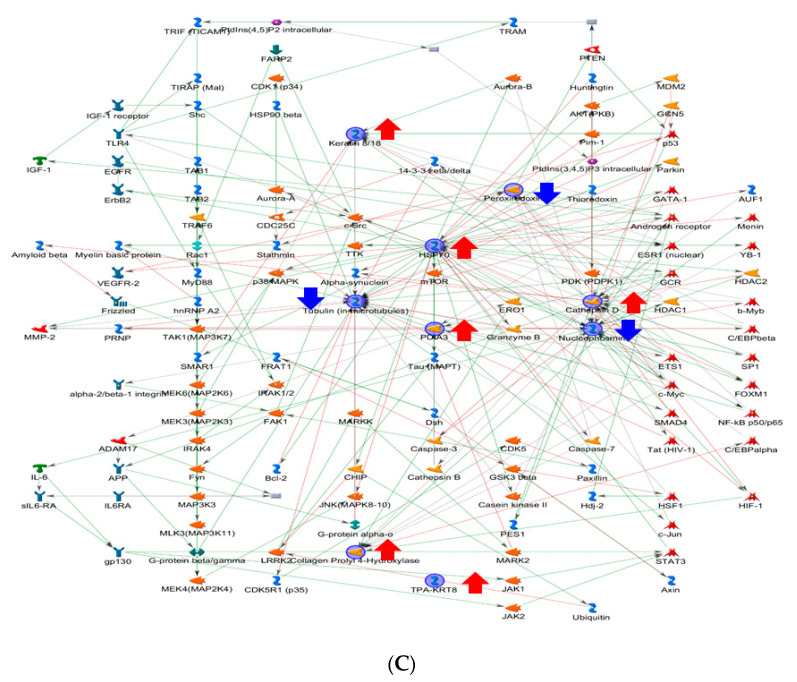
(**A**) Silver-stained 2-DE patterns of MCF-7 and ZR-75-1 cells. The protein lysate (180 μg) was focused on a pH 4–7 linear IPG strip before being separated on 10% polyacrylamide gels and the protein spots with significantly increasing intensity were labeled as Arabic numerals. (**B**) Validation of the difference in protein expression revealed by 2-DE experiment between MCF-7 and ZR-75-1 cells. Protein levels of PDIA3, cathepsin D, Nucleophosmin and PRX2 were assessed by a Western blot analysis. The quantified results were calculated and indicated by the bar chart. GAPDH was used as a loading control (*** *p* < 0.001). (**C**) Biological network analyses of differentially expressed proteins using MetaCore^TM^ mapping tools. Nodes represent proteins and lines between the nodes indicate direct protein–protein interactions. The red up arrows demonstrate the increased proteins and the blue down arrows indicate the decreased proteins. The various proteins on this map are indicated by different symbols representing the functional class of the proteins.

To further reveal the particular proteins and potential mechanisms involved in the regulation of relapse potential in different luminal subtypes of breast cancer, 2-DE analysis, together with MALDI-MS, was performed to comprehensively explore the global protein changes with both high sensitivity and efficacy. Proteins extracted from MCF-7 and ZR-75-1 cells were separated by 2-DE gels and the representative set of silver-stained gels from reproducible gel patterns of three independent experiments are presented in Figure 2A. Approximately 860 protein spots appeared in each gel and MS analysis unambiguously identified the 11 proteins labeled with Arabic numerals with more than 1.5-fold changes in protein volume as summarized in Table 1. We then selected the meaningfully-changed proteins that played critical roles in the modulation of cancer progression for the Western blotting assay. In line with the 2-DE results, a significant increasing tendency in the expression of Nucleophosmin and PRX2 was verified in the MCF-7 cell; otherwise, the ZR-75-1 cell showed marked upregulation in the amount of the cathepsin D and PDIA3 protein with respect to the MCF-7 cell (Figure 2B).

### 2.3. Functional Network Analysis

**Figure 3:**
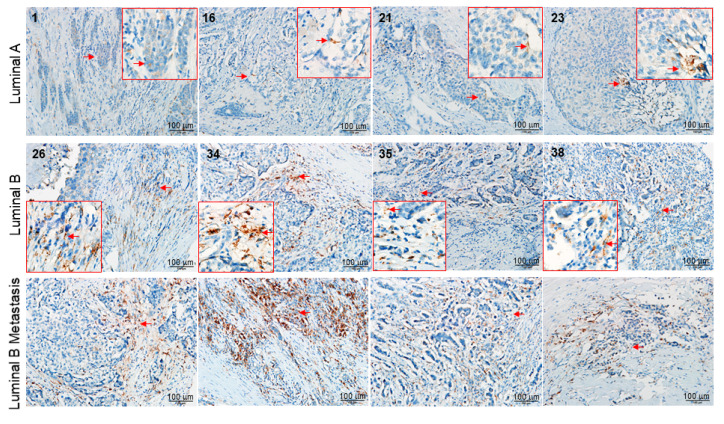
Immunohistochemical study of cathepsin D expression in luminal A, B subtypes of breast cancer tissues and metastastic lymph nodes obtained from luminal B subtype. The positive signal was presented in brown color and indicated by red arrows, which was zoomed in and shown by red square.

Next, MetaCore^TM^ analytical software was used to predict the relationship of the targeted proteins revealed by the proteomic analysis and the underlying pathways linked to the relapse property in the luminal B subtype of breast cancer. As shown in Figure 2C, the protein–protein interaction networks indicated that the target proteins, including cathepsin D and B23, were mainly associated with cell differentiation and apoptosis, while the recurrence of breast cancer majorly led to uncontrolled proliferation triggered by oncogenes such as c-Myc. The algorithm was used to build biological networks from the uploaded proteins and assign a biological process to each network. Furthermore, the in situ expression of cathepsin D was further confirmed by IHC of the clinical samples, in which cathepsin D was highly expressed in the luminal B subtype compared to that of the luminal A subtype. In addition, more advanced metastasis was observed in the patients with the luminal B subtype, which was manifested by the invasion of cancer cells in the lymph nodes and the extensive expression of cathepsin D, suggesting that the luminal B subtype could warrant the subsequent malignance of breast cancer. The IHC results of selected samples were demonstrated in Figure 3 and the clinical information was listed in Supplementary Table S1.

### 2.4. Identify the Cathepsin d-Dependent Regulation of Proteins in ZR-75-1 Cells

**Figure 4:**
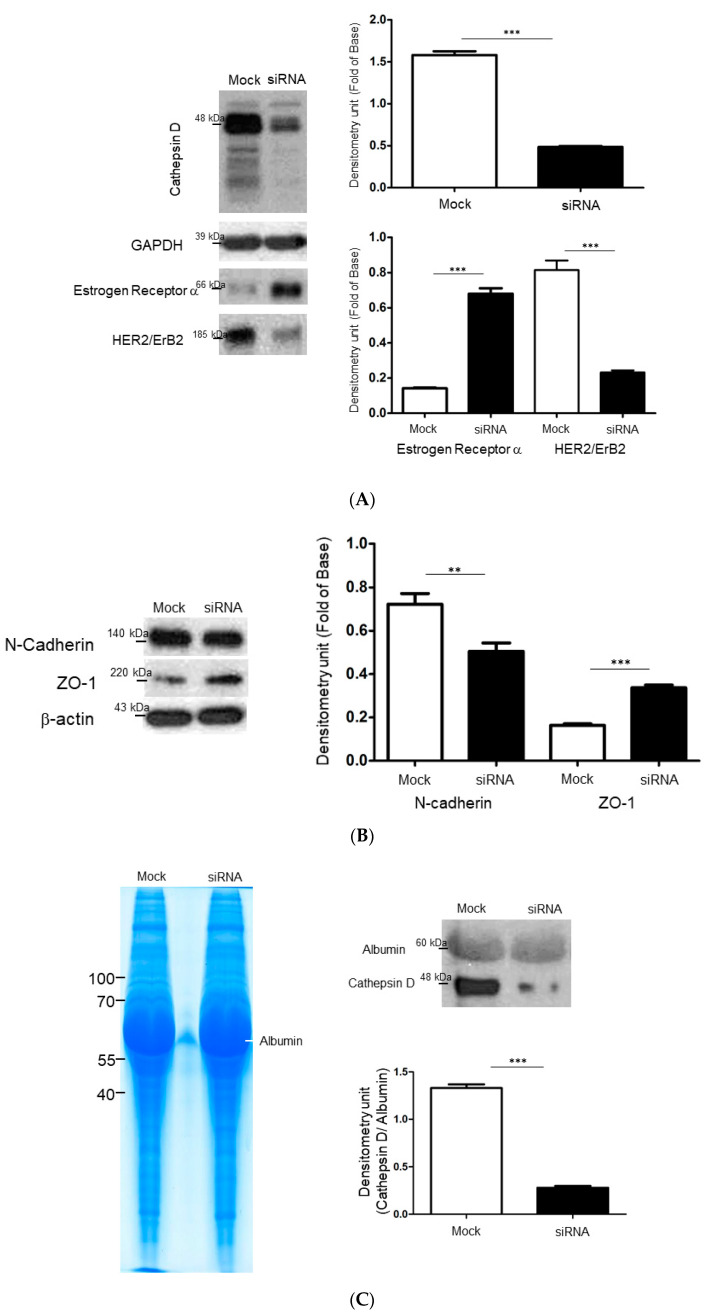

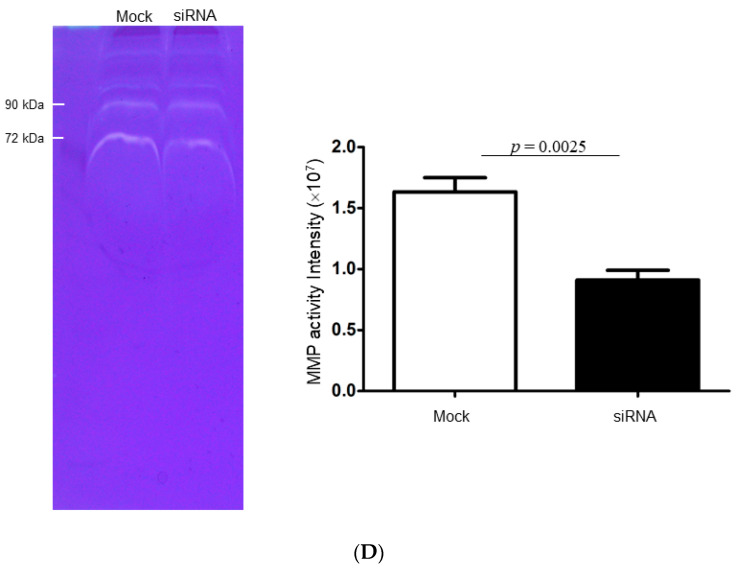
(**A**) Confirmation of changes in protein expression under RNA interference-mediated silencing of cathepsin D treatment. Protein levels of cathepsin D, ER and HER2 were assessed by the Western blot analysis. The intensity was calculated using the Image Pro-Plus 4.5 computer program and indicated by the bar chart. (**B**) The levels of cathepsin d-modulated EMT associated proteins including ZO-1 and N-cadherin were evaluated by the Western blot analysis. The quantified results were demonstrated by the bar chart (** *p* < 0.01; *** *p* < 0.001) and β-actin was utilized as a loading control. (**C**) PVDF membrane stained with Coomassie blue R-250 was applied to visualize loading amount of proteins (left panel) and validation of changes in protein expression in condition medium samples after RNA interference-mediated silencing of cathepsin D (right panel). (**D**) Gelatin zymography was performed with condition media of ZR-75-1 cells with or without silencing cathepsin D transfection. Relative matrix metalloproteinases (MMPs) were quantified and presented as bar charts. The students’ *t*-test was applied for comparison between 2 groups compared to the mock.

The above results implied that cathepsin D is a key factor involved in the relapse of breast cancer and could eventually regulate EMT by means of modulating other associated proteins. Western blotting was used to affirm the protein changes related to the molecular characteristics and EMT progression with or without transient cathepsin D silencing in the ZR-75-1 cells. At first, the silence of cathepsin D obviously inhibited the level of HER2 and markedly stimulated the expression of ER, which entirely altered the original molecular features of the ZR-75-1 cells (Figure 4A). We further detected significant increases in the levels of ZO-1, but N-cadherin showed remarkable downregulation after the diminishment of cathepsin D, as compared to the mock (Figure 4B), suggesting that cathepsin D may promote EMT initiation and induce the relapse of breast cancer cells. The above results implied that cathepsin D is strongly involved in cell migration and invasion as an organizer. Western blotting was then conducted to analyze the medium samples with or without cathepsin D diminishment. Under optimized conditions, a more than 80% reduction in the level of cathepsin D was confirmed, and the loading control for the Western blotting indicated that an equal amount of protein was applied (Figure 4C). We next analyzed whether cathepsin D silencing affected the generation of MMPs. Zymographic assays demonstrated that cathepsin D siRNA significantly inhibited total MMP activity in the siRNAs-transfected cells (Figure 4D).

## 3. Discussion

Breast cancer is a heterogeneous carcinoma that may recur several years after the initial diagnosis and treatment. Intrinsic breast cancer subtypes are currently defined by a combination of morphologic, genomic, and proteomic features, which benefits the diagnosis, prognosis, and treatment strategies for predicting the risk of recurrence in the clinic. However, many patients develop drug resistance, breast cancer relapse, or therapy failure. The most rational explanation for such scenarios is that the biologic heterogeneity of breast cancer subtypes is not thoroughly reflected by clinical parameters and pathological markers [17,18]. Especially, the luminal A and luminal B subtypes exhibit different risks in breast cancer relapse, while the integration of proteomics and network analysis in the pathways has not been fully evaluated and reported. Therefore, we explored potent protein markers for identifying the important mechanisms involved in breast cancer progression and for providing better treatment strategies upon breast cancer.

The high-throughput dataset generated by 2-DE analysis combined with MALDI-TOF-MS was performed to comprehensively delineate the global protein profiles reflecting the disparities between these two breast cancer subtypes. Of note, 11 protein spots displaying significant and meaningful changes were verified. The protein abundance changes were highly indicative of specific cell responses, including epithelial-to-mesenchymal transition (EMT), tumorigenesis, apoptosis and inflammation. Selected proteins that could be pivotal to breast cancer relapse are listed as follows.

Cytokeratins are known to play pivotal roles in cellular growth, motility, and signaling. It has been well documented that CK19 modulates ER stress signaling, resulting in survival and dormancy in breast cancer cells [19]. Herein, elevated CK19 level, which is considered an independent prognostication indicator in cancer patients, was identified in the ZR-75-1 cells. The high expression of CK19 in the luminal B subtype cancer cells implicated its potential role in the improvement of tailored treatments in the future.

Peroxiredoxin 2 is a redox regulatory protein that plays an important role in maintaining ROS homeostasis in the tumor microenvironment by coupling with the thioredoxin/thioredoxin reductase system. It is particularly downregulated in metastatic malignant skin cancers, such as melanoma [20]. Decreased level of peroxiredoxin 2 causes the accumulation of intracellular ROS, which in turn results in the disruption of e-cadherin/β-catenin complexes and leads to the promotion of melanoma cell migration and metastasis [21]. In this regard, the reduced expression of peroxiredoxin 2 among the differentially-expressed proteins in the ZR-75-1 cell line implies the high risk of relapse and metastasis for the luminal B subtype of breast cancer.

The 2-DE data also revealed that nucleophosmin, an estrogen regulated protein, was obviously downregulated in the ZR-75-1 cell with respect to the MCF-7 cell. Epithelial cells of histologically normal breast displayed high levels of nucleophosmin, yet the overproduction of nucleophosmin in the MDA-MB-231 breast cancer cells abolished their growth, thereby supporting a tumor suppressive role of nucleophosmin in breast cancer [22]. We therefore suggest that reduced levels of B23 protein associated with hormonal regulation and chemotherapeutic response could be considered as an independent prognostic factor of poor prognosis in breast cancer.

Specifically, cathepsin D was overexpressed and abundantly secreted by the ZR-75-1 cells, which was also in line with the results derived from histological research. According to previous investigations, the role of cathepsin D in breast carcinoma is significant [23,24,25,26]. It has been reported to have autocrine properties and a number of mechanisms involving cathepsin D in the modulation of the extracellular matrix (ECM), thus facilitating the release of ECM-bound fibroblast growth factors and encouraging further tumor growth, angiogenesis, and cancer relapse. Therefore, we particularly focused on the functional roles of cathepsin D involved in the progression of different breast cancer subtypes. Consistent with the above data, cathepsin D knockdown could also significantly reduce the MMP activity, which is essential for the migration of cancer cells. These findings supported the well-established positive correlation between cathepsin D overexproduction and poor overall survival. Again, increased levels of cathepsin D in primary and metastatic tumors in luminal B subtype have revealed that the cathepsin D expression pattern is a critical biological cell event associated with local recurrence and metastasis. Most importantly, we observed that cathepsin D silence induced tremendous changes in the expression of ER and HER2 in ZR-75-1 cells, which have the similar characteristics to MCF-7 cells. As previously reported, estrogen in ER-positive breast cancer stimulates tumor growth but inhibits invasion and motility [27,28]. This is consistent with our finding that the ER-rich MCF-7 cells showed more proliferation than the ZR-75-1 cells, suggesting that cathepsin D may control ER-independent progression of breast cancer. As expected, cathepsin D silencing also stimulated the expression of ZO-1 and suppressed the level of N-cadherin, demonstrating that cathepsin D, as an independent marker of poor prognosis, could activate EMT, and in turn, induce cancer relapse and metastasis in luminal B subtype patients [29].

We also explored the differential regulation of cathepsin D isoforms via proteome tools. Our data showed a significant increase of several acidic cathepsin D isoforms between pH values of 4 to 6 in the ZR-75-1 cells. Several studies have found that the apparent increased relative amounts of acidic cathepsin D isoforms are coupled with malignant breast tissue due to the abnormal glycosylation of cathepsin D [30]. Additionally, the inactive procathepsin D in the acidic compartment is required to be proteolytically active. We concluded that the immersion of cathepsin D with an acidic pH in the hypoxic regions of solid tumors conduces a proteolytic cascade that accelerates cancer cell invasion and metastasis.

Taken together, cathepsin D can modulate the expression of HER2, as well as ER, and influence the ability of proliferation, survival, invasion, and metastasis in the luminal breast cancer cells. The expression level of cathepsin D could be a crucial marker in making treatment decisions upon patients with ambiguous luminal A or B breast cancer consequently.

## 4. Conclusions

**Figure 5:**
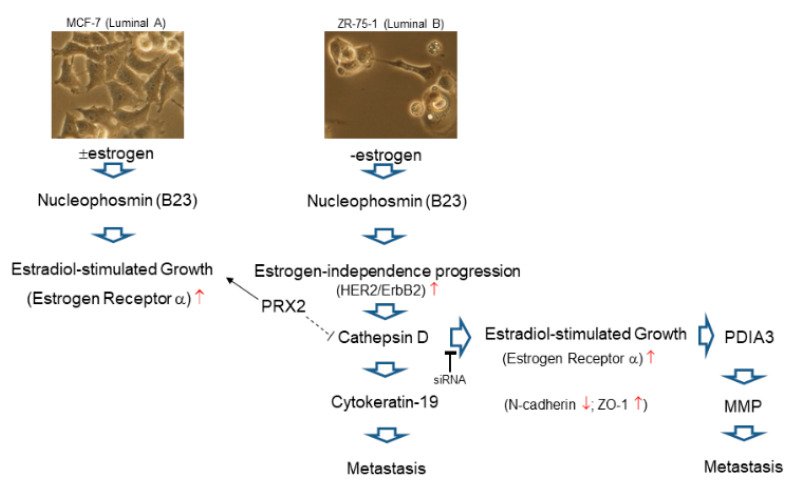
Schematic diagram of cathepsin d-mediated regulation in ECM and EMT, eventually resulting in breast cancer relapse and metastasis (red upward arrows mean increase and red downward arrows indicate decrease).

Our studies demonstrated that a panel of biomarkers including cathepsin D, CK19, nucleophosmin and peroxiredoxin 2 would be applied as a set of indicators for breast cancer relapse, and they could also represent potential therapeutic targets under sufficient validation. Integrative study including previous documents, functional proteome results and bioinformatics findings, nucleophosmin involved in modulating cell growth is induced in estrogen-dependent MCF-7 cell (luminal A subtype) while progression of ZR-75-1 cell (luminal B subtype) could attribute to estrogen-independent pathway where cathepsin D would affect redox regulatory protein, cytoskeleton, hormone receptors and chaperones, which in turn, promote the long-term possibility for cancer relapse. We further showed that the knockdown of cathepsin D altered the expression of ER, which significantly contributes to the attenuation of drug resistance and metastatic potential via suppressing PDIA3 as well as MMPs. (Figure 5). Finally, our findings suggest that global proteome profiling is a feasible tool to unveil cancer biosignatures for tumor subtyping and predicting the risk of recurrence for clinical use.

## 5. Materials and Methods

### 5.1. Cell Culture

MCF-7 and ZR-75-1 cells were purchased from Bioresource Collection and Research Center (Food Industry Research and Development Institute, Taiwan) and grown in culture flasks (Nunc, Roskilde, Denmark) containing RPMI1640, with 10% heat-inactivated fetal bovine serum (FBS) and streptomycin sulfate, in a 5% CO_2_ atmosphere at 37 °C until cells were sub-confluent. After incubation, morphological comparison between MCF-7 and ZR-75-1 cells was observed by using the Olympus microscope (IX71) at 20× fitted with an ocular grid.

### 5.2. Two-Dimensional Electrophoresis (2-DE) and Image Analysis

2-DE is a powerful tool to analyze differential protein expression levels between MCF-7 and ZR-75-1 cells with both high sensitivity and efficacy. The cell pellet was then solubilized in lysis buffer containing 7 M urea, 2 M thiourea, 4% CHAPS, 65 mM DTT, 1 mM PMSF and protease inhibitor cocktail (AMRESCO, Solon, OH, USA) on ice and subjected to sonication for 10 s. The lysate was centrifuged at 10,000 rpm (Kubota, Osaka, Japan) at 4 °C for 30 min to remove insoluble material. The concentration of supernatant was measured by using the Bradford Protein Assay Kit (AMRESCO). Protein extracts (180 μg) were applied to 18-cm IPG linear strip (GE Healthcare, Göteborg, Sweden,) and separated on the IPGphor III System for the first dimension. The IPG strips were equilibrated in a solution containing 50 mM Tris–HCl (pH 8.8), 6 M urea, 2% SDS, 30% glycerol and 2% DTT followed by treatment of the same solution except that DTT was replaced with 2.5% iodoacetamide. The 2-DE was carried out on 10% acrylamide gels (Bio-Rad, Hercules, CA, USA) and the gels were visualized by silver staining. Protein spots were quantified using the Prodigy SameSpots software (Nonlinear Dynamics, Newcastle, UK). Each spot intensity volume (%) was determined by background subtraction and total spot volume normalization; the resulting spot volume percentage was used for comparison between groups. More than 1.5-fold alterations at 95% confidence interval (*p* < 0.05) were considered as statistically significant [31]. All experiments were repeated three times to confirm the reproducibility.

### 5.3. In-Gel Digestion of Proteins and Mass Spectrometric Analysis

Spots of interest were excised and in-gel digested with trypsin for further protein identification according to previously described procedures [32]. After digestion, the tryptic peptides were acidified with 0.5% TFA and loaded onto an MTP AnchorChip^TM^ 600/384 TF (Bruker-Daltonik, Bremen, Germany). MS analysis was performed on an Ultraflex^TM^ MALDI-TOF mass spectrometer (Bruker-Daltonik). Monoisotopic peptide masses were assigned and used for database searches with the MASCOT search engine (Matrix Science, London, UK). Search parameters were set as follows: a maximum allowed peptide mass error of 50 ppm, and consideration of one incomplete cleavage per peptide.

### 5.4. Biological Network Analysis Using MetaCore^TM^

To further explore the relationship of differentially expressed proteins revealed by the proteomics analysis and their significance in the mechanisms linked to recurrence of breast cancer, we applied MetaCore^TM^ software (vers. 5.2 build 17389, GeneGo, St. Joseph, MI, USA) to reveal associated ontological classes and relevant pathways [31]. The algorithm builds biological networks from uploaded proteins and assigns a biological process to each network.

### 5.5. Tissue Array

Commercial tissue array was utilized to validate the expression level and pattern of cathepsin D in clinical samples. One tissue column (2.0 mm each in diameter) was obtained from each selected paraffin block and arranged in separate new paraffin blocks with 60 holes by using a trephine apparatus (SuperBioChips Laboratories, Seoul, Korea). These microarray blocks were sectioned at 4 μm thickness and processed for immunohistochemical staining. The sections were rehydrated with graded ethanol and immersed in Tris-buffered saline after removal of paraffin with xylene [33]. IHC with a cathepsin D antibody (Santa Cruz Biotech. Dallas, TX, USA; 1:200-diluted in phosphate-buffered saline (PBS)) was applied. Next, sections were counterstained with Mayer’s hematoxylin for 2 min, and slides were evaluated under a light microscope (Olympus BX51, Tokyo, Japan). Digital photomicrographs were then processed with DP-72 (Olympus).

### 5.6. Western Blot Analysis

Western blot analysis was applied to perform and quantify the amount of protein. The protein obtained from the skin was isolated using 1× cell lysis buffer (Cell Signaling) and the concentration was determined with the Bradford Protein Assay Kit (AMRESCO). The specific antibodies used in the current study were listed as follows: PDIA3, Nucleophosmin, ER, HER2, ZO-1, N-cadherin (Cell Signaling), GAPDH, PRX2, cathepsin D (Santa Cruz), and β-actin (Abcam, Cambridge, UK). The band intensity was quantified by using GeneTools software (Syngene, Cambridge, UK) and the level of GAPDH or β-actin was performed as internal control [34,35]. All experiments were performed in biological triplicate to confirm the reproducibility.

### 5.7. Gene Silencing by Small Interfering RNA

In the study, knockdown of cathepsin D was achieved by small interfering RNA to evaluate the function of cathepsin D in modulation of proteins or molecules associated with recurrence of breast cancer. ZR-75-1 cells were plated onto 6-well plates (1 × 10^5^ cells/well), maintained in antibiotic-free medium for 24 h, and transfected with a mixture containing Opti-MEM, 8 μL/well Lipofectamine 2000 (Invitrogen, San Diego, CA, USA), and either 0.5 μg/well scrambled siRNA (mock) or a mixture of 3 cathepsin D siRNAs for 6 h [31]. The sequences of these siRNAs are available from the manufacturer. At 48 h post-transfection, cells were harvested and subjected to Western blot analyses. All experiments were performed in biological triplicate to confirm the reproducibility.

### 5.8. Gelatin Zymography

Zymographic assays provide a reliable assessment in human cancer progression. Media with or without siCathepsin D treatment were collected, and gelatin zymography was performed as previously described [31]. After electrophoresis, gels were washed with 50 mM Tris–HCl, at pH 7.4, containing 2.5% Triton X-100 (*v*/*v*) for 1 h, then incubated at 37 °C for overnight in 50 mM Tris–HCl buffer containing 5 mM CaCl_2_. Digestion was terminated, and gels were stained with 0.5% Coomassie brilliant blue R250, followed by destaining with 10% acetic acid and 10% methanol. Enzyme-digested regions were observed as white bands against a blue background. Zones of enzymatic activity were seen as negatively stained bands.

### 5.9. Statistical Analysis

The statistical analysis was executed with Prism software (v5.0, Prism GraphPad, San Diego, CA, USA). The Student’s t-test was applied for comparison between 2 groups and one-way analysis of variance (ANOVA) was used for multiple groups (≥3 groups) comparison. All data in this study were obtained from at least 3 individual experiments and presented as mean ± standard deviation (SD). The *p*-value <0.05 was considered to be statistically different.

## Figures and Tables

**Table 1:** List of differentially expressed proteins between ZR-75-1 and MCF-7.

Spot No.	Protein Name	Accession Number	Mw/pI	Score (Coverage) ^1^	Match Fragment	Fold Change ^2^ (ZR-75-1 vs. MCF-7)	*p*-Value ^3^	Function
1	Peroxiredoxin-2	P32119	22.049/5.66	111 (56%)	15	−3.59 ± 0.04	0.001	Plays a role in cell protection against oxidative stress by detoxifying peroxides and as sensor of hydrogen peroxide-mediated signaling events.
2	Heat shock protein beta-1	P04792	22.826/5.98	71 (55%)	16	−1.56 ± 0.08	0.045	Plays a role in stress resistance and actin organization.
3	Cathepsin D	P07339	45.037/6.10	85 (39%)	17	+5.88 ± 0.02	0.001	Involved in the pathogenesis of several diseases such as breast cancer and possibly Alzheimer disease.
4	Nucleophosmin (B23)	P06748	32.7264.14	20(11%)	3	−2.46 ± 0.03	0.038	Involved in diverse cellular processes such as ribosome biogenesis, centrosome duplication, protein chaperoning, histone assembly, cell proliferation, and regulation of tumor suppressors p53/TP53 and ARF.
5	Cytokeratin-19	P08727	44.079/5.04	255 (78%)	38	+1.58 ± 0.03	0.002	Together with KRT8, helps to link the contractile apparatus to dystrophin at the costameres of striated muscle.
6	Tubulin beta chain	P07437	50.096/4.75	108 (44%)	22	−1.68 ± 0.01	0.049	Tubulin is the major constituent of microtubules.
7	Tubulin α-1B chain	P68363	50.804/4.94	50 (34%)	11	−1.57 ± 0.05	0.042	Tubulin is the major constituent of microtubules.
8	Cytokeratin-8	P05787	53.529/5.52	170 (50%)	31	+3.28 ± 0.04.	0.003	Together with KRT19, helps to link the contractile apparatus to dystrophin at the costameres of striated muscle.
9	Protein disulfide-isomerase A3	P30101	55.328/6.42	86 (45%)	19	+2.58 ± 0.01	0.018	Catalyzes the rearrangement of -S-S- bonds in proteins.
10	Protein disulfide-isomerase	P07237	57.48/4.76	70 (45%)	23	+2.35 ± 0.04	0.023	This multifunctional protein catalyzes the formation, breakage and rearrangement of disulfide bonds.
11	Heat shock cognate 71 kDa protein	P11142	71.082/5.37	136 (50%)	32	+3.45 ± 0.04	0.029	Molecular chaperone implicated in a wide variety of cellular processes, including protection of the proteome from stress, folding and transport of newly synthesized polypeptides, activation of proteolysis of misfolded proteins and the formation and dissociation of protein complexes.

^1^ Coverage is defined as the ratio of the protein sequence covered by matched peptides to the full length of the protein sequence. SwissProt 2020_02 (562253 sequences; 202,348,262 residues). ^2^ The fold changes of protein between ZR-75-1 and MCF-7. “+” mean upregulation and “−” indicated downregulation of protein volume by analyzing the gel images with Prodigy SameSpots^TM^ software. ^3^ *p*-value were produced by Prodigy SameSpots^TM^ software. *p* < 0.005 was considered significant for the differences.

## Supplementary Materials

The following are available online at https://www.mdpi.com/1422-0067/21/17/6077/s1. Table S1.

## Abbreviations

ER	estrogen receptor
HER2	human epidermal growth factor receptor 2
2-DE	Two-dimensional electrophoresis
MALDI-TOF-MS	Matrix-Assisted Laser Desorption/Ionization Time of Flight Mass Spectrometry
PMF	Peptide mass fingerprinting
EMT	epithelial-to-mesenchymal transition
IPG	Immobilized pH gradient
IHC	Immunohistochemistry
SDS-PAGE	Sodium dodecyl sulfate-polyacrylamide gel electrophoresis
TBST	Tris-buffered saline Tween-20
PVDF	polyvinylidene difluoride
ECM	extracellular matrix
MMP	matrix metalloproteinase
PRX2	peroxiredoxin 2

## References

[1] Voduc K.D., Cheang M.C., Tyldesley S., Gelmon K., Nielsen T.O., Kennecke H. (2010). Breast cancer subtypes and the risk of local and regional relapse. J. Clin. Oncol..

[2] Li Z.H., Hu P.H., Tu J.H., Yu N.S. (2016). Luminal B breast cancer: Patterns of recurrence and clinical outcome. Oncotarget.

[3] Abubakar M., Sung H., Bcr D., Guida J., Tang T.S., Pfeiffer R.M., Yang X.R. (2018). Breast cancer risk factors, survival and recurrence, and tumor molecular subtype: Analysis of 3012 women from an indigenous Asian population. Breast Cancer Res..

[4] Calhoun B.C., Collins L.C. (2015). Predictive markers in breast cancer: An update on ER and HER2 testing and reporting. Semin. Diagn. Pathol..

[5] Beca F., Polyak K. (2016). Intratumor Heterogeneity in Breast Cancer. Adv. Exp. Med. Biol..

[6] Weigelt B., Geyer F.C., Reis-Filho J.S. (2010). Histological types of breast cancer: How special are they?. Mol. Oncol..

[7] Rivenbark A.G., O’Connor S.M., Coleman W.B. (2013). Molecular and cellular heterogeneity in breast cancer: Challenges for personalized medicine. Am. J. Pathol..

[8] Yap Y.S., Lu Y.S., Tamura K., Lee J.E., Ko E.Y., Park Y.H., Cao A.Y., Lin C.H., Toi M., Wu J. (2019). Insights into Breast Cancer in the East vs. the West: A Review. JAMA Oncol..

[9] Yu N.Y., Iftimi A., Yau C., Tobin N.P., van ‘t Veer L., Hoadley K.A., Benz C.C., Nordenskjöld B., Fornander T., Stål O. (2019). Assessment of Long-term Distant Recurrence-Free Survival Associated with Tamoxifen Therapy in Postmenopausal Patients With Luminal A or Luminal B Breast Cancer. JAMA Oncol..

[10] Palomeras S., Ruiz-Martínez S., Puig T. (2018). Targeting Breast Cancer Stem Cells to Overcome Treatment Resistance. Molecules.

[11] Byler S., Goldgar S., Heerboth S., Leary M., Housman G., Moulton K., Sarkar S. (2014). Genetic and epigenetic aspects of breast cancer progression and therapy. Anticancer Res..

[12] Huang J., Li H., Ren G. (2015). Epithelial-mesenchymal transition and drug resistance in breast cancer (Review). Int. J. Oncol..

[13] Veeraraghavan J., Ma J., Hu Y., Wang X.S. (2016). Recurrent and pathological gene fusions in breast cancer: Current advances in genomic discovery and clinical implications. Breast Cancer Res. Treat..

[14] Wood S.L., Westbrook J.A., Brown J.E. (2014). Omic-profiling in breast cancer metastasis to bone: Implications for mechanisms, biomarkers and treatment. Cancer Treat. Rev..

[15] Parsons J., Francavilla C. (2020). ‘Omics Approaches to Explore the Breast Cancer Landscape. Front. Cell Dev. Biol..

[16] Osin P., Shipley J., Lu Y.J., Crook T., Gusterson B.A. (1998). Experimental pathology and breast cancer genetics: New technologies. Recent Results Cancer Res..

[17] Naser Al Deen N., Nassar F., Nasr R., Talhouk R. (2019). Cross-Roads to Drug Resistance and Metastasis in Breast Cancer: miRNAs Regulatory Function and Biomarker Capability. Adv. Exp. Med. Biol..

[18] Mueller C., Haymond A., Davis J.B., Williams A., Espina V. (2018). Protein biomarkers for subtyping breast cancer and implications for future research. Expert Rev. Proteom..

[19] Bambang F., Lu D., Li H., Chiu L.L., Lau Q.C., Koay E., Zhang D. (2009). Cytokeratin 19 Regulates Endoplasmic Reticulum Stress and Inhibits ERp29 Expression via p38 MAPK/XBP-1 Signaling in Breast Cancer Cells. Exp. Cell Res..

[20] Carta F., Demuro P.P., Zanini C., Santona A., Castiglia D., D’Atri S., Ascierto P.A., Napolitano M., Cossu A., Tadolini B. (2005). Analysis of candidate genes through a proteomics-based approach in primary cell lines from malignant melanomas and their metastases. Melanoma Res..

[21] Lee D.J., Kang D.H., Choi M., Choi Y.J., Lee J.Y., Park J.H., Park Y.J., Lee K.W., Kang S.W. (2013). Peroxiredoxin-2 represses melanoma metastasis by increasing E-Cadherin/β-Catenin complexes in adherens junctions. Cancer Res..

[22] Karhemo P.R., Rivinoja A., Lundin J., Hyvönen M., Chernenko A., Lammi J., Sihto H., Lundin M., Heikkilä P., Joensuu H. (2011). An Extensive Tumor Array Analysis Supports Tumor Suppressive Role for Nucleophosmin in Breast Cancer. Am. J. Pathol..

[23] Rochefort H., Garcia M., Glondu M., Laurent V., Liaudet E., Rey J.M., Roger P. (2000). Cathepsin D in breast cancer: Mechanisms and clinical applications, a 1999 overview. Clin. Chim. Acta.

[24] Dian D., Heublein S., Wiest I., Barthell L., Friese K., Jeschke U. (2014). Significance of the tumor protease cathepsin D for the biology of breast cancer. Histol. Histopathol..

[25] Dubey V., Luqman S. (2017). Cathepsin D as a Promising Target for the Discovery of Novel Anticancer Agents. Curr. Cancer Drug Targets.

[26] Liaudet-Coopman E., Beaujouin M., Derocq D., Garcia M., Glondu-Lassis M., Laurent-Matha V., Prébois C., Rochefort H., Vignon F. (2006). Cathepsin D: Newly discovered functions of a long-standing aspartic protease in cancer and apoptosis. Cancer Lett..

[27] Rochefort H. (1998). Estrogens, cathepsin D and metastasis in cancers of the breast and ovary: Invasion or proliferation?. C. R. Seances Soc. Biol. Fil..

[28] Rochefort H., Platet N., Hayashido Y., Derocq D., Lucas A., Cunat S., Garcia M. (1998). Estrogen receptor mediated inhibition of cancer cell invasion and motility: An overview. J. Steroid Biochem. Mol. Biol..

[29] Chu P.Y., Hou M.F., Lai J.C., Chen L.F., Lin C.S. (2019). Cell Reprogramming in Tumorigenesis and Its Therapeutic Implications for Breast Cancer. Int. J. Mol. Sci..

[30] Garcia M., Platet N., Liaudet E., Laurent V., Derocq D., Brouillet J.P., Rochefort H. (1996). Biological and Clinical Significance of Cathepsin D in Breast Cancer Metastasis. Stem Cells.

[31] Pan T.L., Wang P.W., Huang C.C., Yeh C.T., Hu T.H., Yu J.S. (2012). Network analysis and proteomic identification of vimentin as a key regulator associated with invasion and metastasis in human hepatocellular carcinoma cells. J. Proteomics.

[32] Wang P.W., Lin T.Y., Hung Y.C., Chang W.N., Yang P.M., Chen M.H., Yeh C.T., Pan T.L. (2019). Characterization of Fibrinogen as a Key Modulator in Patients with Wilson’s Diseases with Functional Proteomic Tools. Int. J. Mol. Sci..

[33] Yom C.K., Noh D.Y., Kim W.H., Kim H.S. (2011). Clinical significance of high focal adhesion kinase gene copy number and overexpression in invasive breast cancer. Breast Cancer Res. Treat..

[34] Wang P.W., Wu T.H., Lin T.Y., Chen M.H., Yeh C.T., Pan T.L. (2019). Characterization of the Roles of Vimentin in Regulating the Proliferation and Migration of HSCs during Hepatic Fibrogenesis. Cells.

[35] Pan T.L., Wang P.W., Leu Y.L., Wu T.H., Wu T.S. (2012). Inhibitory effects of Scutellaria baicalensis extract on hepatic stellate cells through inducing G2/M cell cycle arrest and activating ERK-dependent apoptosis via Bax and caspase pathway. J. Ethnopharmacol..

