# Supplementary material for: Characterizing the Relapse Potential in Different Luminal Subtypes of Breast Cancers with Functional Proteomics
Source: Int J Mol Sci. 2020 Aug 24;21(17):6077. doi: 10.3390/ijms21176077 (PMC7504407; doi:10.3390/ijms21176077)
Supplement: Supplementary file 1 [file ijms-21-06077-s001.pdf]

Supplement Table

Luminal A

No.	Age	Sex	Organ	Diagnosis	pTNM	ER^+	PR^+	$Her2^+$	Histologic grade
1	59	F	Breast	infiltrating duct carcinoma	T2N0M0	+	+	-	II
16	53	F	Breast	infiltrating duct carcinoma	T2N1miM0	+	+	-	II
21	56	F	Breast	infiltrating duct carcinoma	T2N0M0	+	+	-	III
23	42	F	Breast	infiltrating duct carcinoma	T2N3aM0	+	+	-	II

Luminal B

No.	Age	Sex	Organ	Diagnosis	pTNM	ER^+	PR^+	$Her2^+$	Histologic grade
26	51	F	Breast	infiltrating duct carcinoma	T2N1aM0	+	+	+	III
34	58	F	Breast	infiltrating duct carcinoma	T2N3aM0	-	-	+	III
35	37	F	Breast	infiltrating duct carcinoma	T2N3aM0	-	-	+	III
38	41	F	Breast	infiltrating duct carcinoma	T2N3aM0	-	-	+	III
